# Editorial: The application of sequencing technologies and bioinformatics methods in cancer biology

**DOI:** 10.3389/fcell.2022.1002813

**Published:** 2022-09-06

**Authors:** Geng Chen, Lu Xie, Fangqing Zhao, David P. Kreil

**Affiliations:** ^1^ Stemirna Therapeutics Co., Ltd., Shanghai, China; ^2^ Institute for Genome and Bioinformatics, Shanghai Institute for Biomedical and Pharmaceutical Technologies, Shanghai, China; ^3^ Computational Genomics Laboratory, Beijing Institutes of Life Science, Chinese Academy of Sciences, Beijing, China; ^4^ Department of Biotechnology, Boku University Vienna, Vienna, Austria

**Keywords:** cancer biology, bulk sequencing, single-cell sequencing, bioinformatics analysis, machine learning

Sequencing technologies including bulk and single-cell approaches have been employed broadly to investigate molecular changes and the underlying mechanisms of cancer development, progression, and metastasis (Donoghue et al., 2020; Lei et al., 2021). For instance, whole-genome and whole-exome sequencing technologies allow the identification of diverse genetic alterations (e.g., point mutations, indels, and structural variations) that may contribute to tumorigenesis and tumor growth (Alexandrov et al., 2020; Consortium, 2020; Li et al., 2020). RNA-seq can enable the exploration of up/down-regulated genes or transcripts, the aberrant events of gene fusion, changed alternative splicing, and different RNA editing in cancers compared to corresponding controls (Dehghannasiri et al., 2019; Demircioglu et al., 2019; Group et al., 2020). The advent of single-cell sequencing approaches further provides unprecedented opportunities for dissecting tumor heterogeneity and cellular dynamics (Kinker et al., 2020; Li et al.; Leader et al., 2021). In the era of big cancer genomics data, bioinformatics approaches are crucial for effectively analyzing and interpreting the increasing amount of sequencing data from different perspectives, such as biomarker discovery (Cheong et al., 2022), outcome prediction (Zhou et al., 2022), and disease association elucidation (Xu et al., 2022). The combination of sequencing technologies and related bioinformatics approaches for cancer dissection is effectively deepening our understanding of tumor biology.

Although enormous progress and fruitful results have been achieved for diverse cancers, great efforts are needed to further promote the development of precision medicine. Specifically, the exploration of novel biomarkers and new computational approaches is crucial for better diagnosis, prognosis, and treatment of cancer patients. In this Research Topic on *The Application of Sequencing Technologies and Bioinformatics Methods in Cancer Biology*, we aimed to collect novel findings and methods in the field of cancer biology related to mining bulk and single-cell sequencing data with bioinformatics approaches for illuminating different types of tumors (Figure 1). A total of 21 original research articles were published in this Research Topic, covering the investigation of new cancer-associated biomarkers and potential underlying mechanisms, as well as novel computational strategies for analyzing tumors. We summarize and discuss the main findings of these studies in this editorial.

**FIGURE 1:**
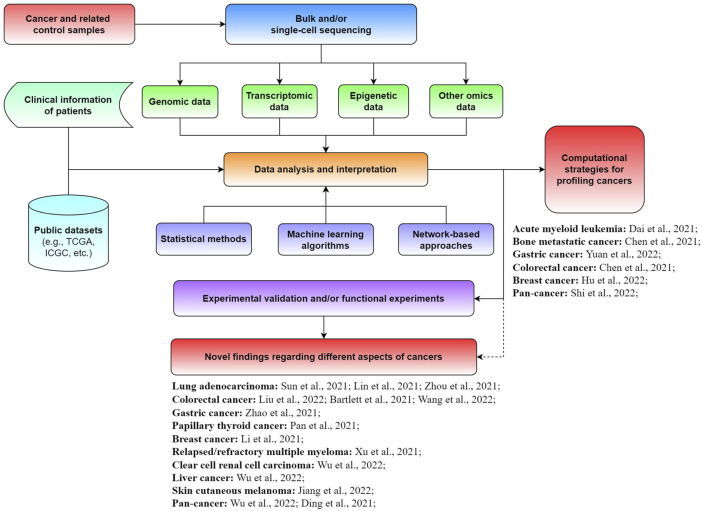
Schematic view of the application of sequencing technologies and bioinformatics approaches for cancer-related studies.

## Biomarker identification and characterization

### Lung cancer

Three studies on this Research Topic dissected lung adenocarcinoma (LUAD) from different perspectives and identified a number of potential prognostic biomarkers. For example, Sun et al. uncovered three ferroptosis subtypes in the LUAD cohort based on expression profiling of ferroptosis driver and suppressor genes. They also detected remarkable differences in terms of the immune microenvironment and biological functions among these three subtypes. A set of ferroptosis-related signatures associated with the prognosis of LUAD patients were identified as well. Their results revealed that ferroptosis could play an important role in LUAD. In another study, Lin et al. investigated LUAD from the view of cellular senescence. They showed that the combination of senescence-related signature (SRS) and immune checkpoint expression or tumor mutation burden was an effective prognostic biomarker for immunotherapy. Their findings highlighted that SRS could regulate the immune microenvironment of LUAD.Zhou et al. explored the immune-oncology profile of LUAD and identified a set of immune genes significantly correlated with progression-free survival. They also found that the risk signature based on several of these immune genes was associated with neutrophil infiltration. Therefore, these three studies screened out different types of prognostic biomarkers for LUAD patients, which could be useful and promising for survival analysis.

### Colorectal cancer

For colorectal cancer (CRC), three studies discovered potential prognostic biomarkers or treatment preditors by analyzing large-scale datasets. For instance, Liu et al. identified five CRC survival-related genes (SLC26A3, GUCA2A, CLCA4, CLCA1, and AQP8) based on hub gene analysis of co-expression and protein-protein interaction networks. They also constructed a promising risk model using three (CLCA1, CLCA4, and GUCA2A) of them for prognostic prediction. Their results indicated that these hub genes could be correlated with CRC development and the survival of patients. Wang et al. constructed a scoring model based on four necroptosis-related genes (FAS, CAMK2B, STAT4, and CYBB), which can effectively predict the prognosis and response of colon cancer patients to chemotherapy and immunotherapy. They also found that the activation of necroptosis-related genes could promote the metastasis of colon cancer. Unlike these two studies, Bartlett et al. investigated the association between miRNAs and immune checkpoint blockade for CRC. They detected a number of miRNAs that were correlated with mutation burden, microsatellite instability, or PD-L1 expression. Among them, three miRNAs (miRNA-146b, miRNA-155, and miRNA-22) were related to the M1 macrophage polarization state and their targets had the potential to impact the TGF-β pathway.

### Other cancer types

In addition to biomarker exploration articles in lung and colorectal cancer, we received articles that explored biomarkers in gastric, thyroid, breast, and other tumor types. For example, Zhao et al. discovered that loss of ARID1A was strongly correlated with the high expression of CD47 in gastric cancer. They identified CD47 as a potential direct downstream target of ARID1A, while the combination of ARID1A and CD47 would be a promising prognostic biomarker. Pan et al. examined the landscape of papillary thyroid cancer (PTC) at single-cell resolution and detected molecular signatures correlated with the disease-free survival of patients. They also found that dendritic cells and B cells could play critical functions in preventing PTC progression. identified GPX1 as a promising prognostic biomarker for breast cancer (Li et al., 2021b). They observed that anhydroicaritin may suppress epithelial-mesenchymal transition by elevating GPX1 expression, which could provide potential guidance for breast cancer treatment. Xu et al. examined the dynamic genomic changes of relapsed/refractory multiple myeloma (RRMM) based on whole-exome sequencing and single-cell RNA-seq data. They found that RUNX3 could be a potential driver and therapeutic target for RRMM. Wu et al. detected KIF23 as an effective prognostic biomarker for clear cell renal cell carcinoma (ccRCC). They also revealed that KIF23 could promote the nuclear translocation of β-catenin, while the knockdown of KIF23 would reduce the proliferation, migration, and invasion of ccRCC. For liver cancer, Wu et al. showed that up-regulated expression of POSTN, LAYN, and HTRA3, and down-regulated expression of AANAT and AFM were associated with poorer overall survival of patients. They also found that the gut microbial metabolites of trimethylamine N-oxide TMAO and POSTN were potential targets for liver cancer treatment. Jiang et al. revealed that TMEM176B could be a diagnostic and prognostic biomarker for skin cutaneous melanoma. The expression of TMEM176B was shown to be correlated with tumor-infiltrating lymphocytes, pathological stage, therapy sensitivity to radiation, as well as tumor ulceration.

Another two studies conducted pan-cancer analyses and detected potential biomarkers for the clinical management of cancers. For example, Wu et al. systematically analyzed AP3S1 in diverse tumor types and found that its expression was widely associated with the immunosuppressive microenvironment. They also demonstrated that AP3S1 could be a pan-cancer biomarker for prognosis and immunotherapy. The other study explored the gene expression profiles of 988 cell lines from 20 distinct cancers by employing several computational methods Ding et al. They identified robust pan-cancer biomarkers for differentiating a variety of cancer types. Overall, the aforementioned studies discovered different types of potential biomarkers for diverse tumors, which could be useful for the diagnosis, prognosis, or treatment of corresponding cancers.

## Computational strategies for profiling cancers

Besides biomarker investigation, several studies on this Research Topic developed new computational strategies for profiling tumors from different aspects. For instance, Dai et al. developed a novel model based on the score of cell type compositions (CTCs) for improving the prognostic analysis of acute myeloid leukemia patients (AML). They further showed that the CTC score could potentially benefit the individualized treatment of AML patients. Chen et al. systematically investigated the chromosome instability (CIN) profile of 280 patients with bone metastatic cancer based on the copy number variations inferred from cell-free DNA (cfDNA) sequencing data. They revealed that CIN quantification with cfDNA provided an effective and non-invasive method for predicting the survival of spine metastasis patients. Yuan et al. developed a scoring approach based on 15-DNA repair gene signatures for effectively predicting the prognosis of gastric cancer patients who received immunotherapies. The scoring system developed by them may benefit the tailored immunotherapy of gastric cancers. Chen et al. proposed an efficient strategy for identifying the circular RNAs (circRNAs) with protein-coding potential in CRC. They also suggested that those circRNAs might be functional in promoting proliferation and invasion ability, while the peptides derived from circRNAs could be potential targets for CRC therapy or diagnosis. Hu et al. designed a panel based on 28 breast cancer-related genes for long-read sequencing (e.g., Oxford Nanopore and Pacific Biosciences platforms). They demonstrated that this approach can effectively detect structural variations in breast cancer patients, which could be used in related clinical investigations. Shi et al. systematically evaluated a gene panel containing ∼1,300 key immuno-oncology genes designed for characterizing tumor microenvironments. Based on the analysis of >1,200 formalin-fixed paraffin-embedded tumor samples, they showed that this panel was comparable with orthogonal platforms (e.g., RNA-seq, hematoxylin and eosin staining, and immunohistochemistry). The computational strategies developed in these studies were useful and promising for exploring and characterizing various aspects of different cancers.

## Summary and perspective

Taken together, the studies published in this Research Topic presented a diversity of interesting and meaningful results for a range of different cancers, which could facilitate our understanding of cancer biology. It is well known that bulk and single-cell sequencing technologies can respectively obtain whole-system and cellular views of tumors. Multi-omics and multimodal strategies are superior to single-omics methods for dissecting cancers since different types of data could be complementary (Li et al., 2021c). Third-generation sequencing technologies are gradually maturing, enabling the production of much longer reads than that of the currently abundant next-generation sequencing protocols (Logsdon et al., 2020; De Coster et al., 2021). Novel computational tools based on advanced machine learning algorithms may further help researchers to process the growing amount of sequencing data more efficiently (Park et al., 2021). These advancements are transforming data analysis and interpretation for better cancer management. Overall, innovation both in sequencing technologies and bioinformatics approaches will continue to facilitate the translation of big cancer genomic data into clinical practice and benefit precision medicine. We hope that this Research Topic will inspire researchers to further investigate cancers by integrative analysis of different omics data in a systematic way.
